# Podocyte specific knockout (KO) of the natriuretic peptide clearance receptor (NPRC) attenuates diabetic kidney disease (DKD)

**DOI:** 10.14814/phy2.70899

**Published:** 2026-05-13

**Authors:** Liming Wang, Yupinp Tang, Qunsheng Dai, Anne F. Buckley, Robert F. Spurney

**Affiliations:** ^1^ Division of Nephrology, Department of Medicine Duke University and Durham VA Medical Centers Durham North Carolina USA; ^2^ Department of Pathology Duke University Medical Center Durham North Carolina USA

**Keywords:** diabetic nephropathy, kidney disease, natriuretic peptides

## Abstract

Glomerular podocytes play a key role in the pathogenesis of diabetic kidney disease (DKD). Recent studies suggest that natriuretic peptides (NPs) are podocyte protective. The beneficial effects of NPs are inhibited by the removal of NPs from the circulation by the NP clearance receptor (NPRC). To determine if inhibiting NP clearance by NPRC ameliorated DKD, we deleted NPRC specifically in glomerular podocytes in a mouse model of type 1 diabetes (Akita mice). We found that diabetes induced a significant increase in albuminuria in WT Akita mice, which was significantly reduced by knockout (KO) of NPRC. Histologic damage was mild in both WT and KO Akita mice and limited to a modest increase in mesangial matrix in both groups. In contrast, expression of the fibrotic markers fibronectin and collagen 1 was significantly increased in isolated glomerular preparations, and expression of both fibrotic markers was significantly improved by podocyte‐specific KO of NPRC. Taken together, these data suggest that inhibiting NP clearance by glomerular podocytes has beneficial effects in a mouse model of early‐stage DKD.

## 1 INTRODUCTION

Diabetic kidney disease (DKD) is a common complication of diabetes (Molitch et al., 2004), and the number of patients with diabetes is increasing worldwide (Maahs et al., 2010; Molitch et al., 2004). While advances have been made in the treatment of DKD over the last several decades (Brenner et al., 2001; Lewis et al., 2001; Packer et al., 2020), DKD remains a common cause of renal failure (Bonnet et al., 2024). As a result, additional treatment approaches are needed to either prevent or slow DKD progression.

Glomerular podocytes are terminally differentiated cells with a limited ability to replicate (Kriz et al., 1998; Wiggins, 2007). These cells play a key role in maintaining the integrity of the glomerular filtration barrier (Jefferson et al., 2008; Wiggins, 2007). Diabetes promotes podocyte injury and damages the glomerular filtration barrier, causing proteinuria. With disease progression, there is a reduction in the number of glomerular podocytes (Dalla Vestra et al., 2003; Meyer et al., 1999; Teiken et al., 2008; Wang et al., 2015), which causes instability and collapse of the glomerular tuft and, in turn, glomerulosclerosis (GS) (D'Agati, 2003).

Previous studies suggest that natriuretic peptides (NPs) have beneficial actions in kidney diseases (Makino et al., 2006; Ogawa et al., 2012; Shen et al., 2016; Staffel et al., 2017; Suganami et al., 2001; Wang et al., 2021; Wang, Tang, et al., 2025). For example, inhibiting NP signaling promoted kidney damage and increased proteinuria in mouse models of glomerular disease (Kato et al., 2017; Ogawa et al., 2012; Staffel et al., 2017). In contrast, overexpression of NPs reduced disease progression in mouse models of DKD (Makino et al., 2006; Suganami et al., 2001). Moreover, recent studies from our lab found that inhibiting degradation of NPs reduced proteinuria and inhibited glomerular injury in a mouse model of focal segmental glomerulosclerosis (FSGS). (Wang, Tang, et al., 2025). While the mechanisms mediating these beneficial effects are not known with certainty, NPs inhibit multiple pathways that play important roles in kidney diseases including RhoA, TGF‐beta, endothelin‐1, calcium signaling, and the renin‐angiotensin system (Francis et al., 2010; Glenn et al., 2009; Kohno et al., 1991; Kurtz et al., 1986; Shen et al., 2016; Shirakami et al., 1993; Tsou et al., 2014). Taken together, these data suggest that enhancing the biologic actions of NPs may be a useful approach to ameliorate kidney diseases.

The effects of NPs are mediated by binding to cell surface receptors and stimulating cGMP generation (Potter et al., 2006). The type A receptor (NPRA) binds both atrial NP (ANP) and brain NP (BNP) (Potter et al., 2006), and the type B receptor (NPRB) binds the C‐type NP (Potter et al., 2006). In contrast, the NP clearance receptor (NPRC) binds and degrades ANP, BNP, and CNP, and negatively regulates the action of NPs (Potter et al., 2006).

Based on these data, we postulated that deleting NPRC specifically in podocytes would enhance local NP levels and reduce podocyte injury in a mouse model of DKD. We found that NPRC knockout (KO) significantly reduced both proteinuria and expression of fibrotic markers in a mouse model of type 1 diabetes (Akita mice). These data suggest that inhibiting NP clearance may be a useful strategy for reducing kidney damage in diabetes.

## 2 RESULTS

### 2.1 Podocyte specific KO of NPRC

**FIGURE 1:**
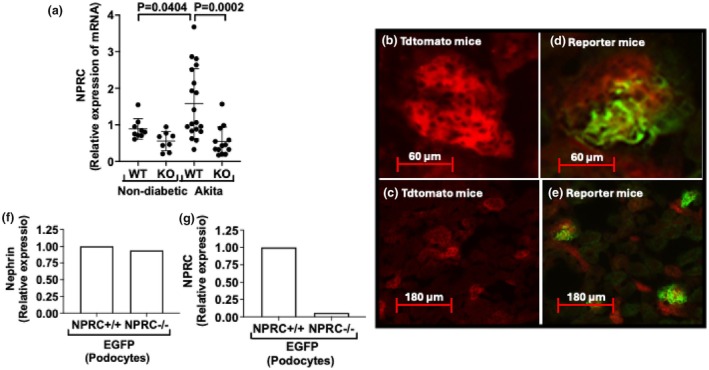
(a) NPRC expression was significantly increased in WT Akita mice compared to non‐diabetic WT mice. There was also a significant decrease in NPRC expression in KO Akita mice compared to WT Akita mice. (b, c) In Tdtomato mice lacking Cre recombinase, Tdtomato was expressed in most cell types without significant expression of EGFP. (d, e) Tdtomato was also widely expressed in both NPRC+/+ and NPRC−/− reporter mice, with EGFP expression localized to podocytes in the glomerular tuft. (f, g) EGFP cells were isolated by flow cytometry and used for quantitative RT‐PCR to assess expression of the podocyte marker nephrin and expression of NPRC. Expression of nephrin was equally expressed in NPRC+/+ and NPRC−/− reporter mice. In contrast, expression of NPRC was difficult to detect in NPRC−/− reporter mice. A total of 49 mice were studied in Figure 1a, and a total of 12 fluorescent pictures were examined in figures b, c, d, and e. For flow cytometry, cortices from 3 to 4 mice were combined to isolate EGFP labeled cells.

We investigated the effects of increasing the podocyte protective actions of NPs in DKD by creating C57BL/6 Akita mice lacking NPRC specifically in glomerular podocytes (KO Akita mice). To determine the effectiveness of NPRC KO, we first evaluated the expression of NPRC mRNA in enriched glomerular preparations from Akita mice using Q‐RT‐PCR (quantitative reverse transcription‐polymerase chain reaction). As shown in Figure 1a, there was a significant increase in the expression of NPRC in wild type (WT) Akita mice compared to non‐diabetic WT mice. In addition, there was a significant decrease in NPRC expression in KO Akita mice compared to WT Akita mice. A similar pattern was observed in the non‐diabetic WT and KO mice, but the difference was not statistically different.

To further examine the effectiveness of NPRC deletion, NPRC KO mice were crossed with reporter mice (Muzumdar et al., 2007) expressing a 2‐color reporter allele (reporter mice). The reporter allele expresses a cell membrane‐localized red fluorescence protein (Tdtomato) with widespread expression in all tissues and cell types prior to Cre‐mediated recombination (Wang et al., 2017). Expression of Cre recombinase induces a cell membrane‐localized enhanced green fluorescence protein (EGFP). (Muzumdar et al., 2007). For the studies, Tdtomato reporter mice were crossed with mice expressing both a podocyte specific Cre‐recombinase (see methods) and LoxP sites flanking exon 3 of NPRC to create either: (1) Tdtomato mice expressing a podocyte specific Cre recombinase (NPRC+/+ reporter mice) or (2) Tdtomato mice expressing both a podocyte specific Cre recombinase and LoxP sites flanking exon 3 of NPRC (NPRC−/− reporter mice). In these mice, both WT and KO reporter mice are labeled with EGFP, but NPRC is deleted only in KO reporter mice.

To determine the effectiveness of NPRC KO, we first examined frozen sections from Tdtomato mice lacking Cre recombinase (Figure 1b,c). In these mice, Tdtomato was widely expressed in most cell types without significant expression of EGFP. We next crossed Tdtomato mice with mice expressing both the podocyte specific Cre‐recombinase and LoxP sites flanking exon 3 of NPRC to create either: (1) Tdtomato mice expressing a podocyte specific Cre recombinase (NPRC+/+ reporter mice) or (2) Tdtomato mice expressing both a podocyte specific Cre recombinase and LoxP sites flanking exon 3 of NPRC (NPRC−/− reporter mice). In both these reporter mice, Tdtomato was widely expressed in most cell types with EGFP expression localized to the glomerular tuft (Figure 1d,e). Given that podocytes are found in the glomerular tuft, these data are consistent with the expression of EGFP in podocytes.

To determine the effectiveness of NPRC KO in the reporter mice, we used flow cytometry to collect EGFP expressing cells from NPRC+/+ and NPRC−/− reporter mice. We then examined the reporter mice for expression of both NPRC and the podocyte marker nephrin. As shown in Figure 1f expression of nephrin was similar in NPRC+/+ and NPRC−/− reporter mice. In addition, NPRC was expressed in NPRC+/+ reporter mice. In contrast, little expression of NPRC was detected in EGFP labeled podocytes from NPRC−/− reporter mice. These data suggest that: 1. EGFP labeled cells express the podocyte specific marker nephrin in the correct anatomical location in the glomerular tuft, and 2. NRC was effectively deleted in podocytes in NPRC−/− reporter mice (Figure 1f,g).

### 2.2 Glucose levels and albuminuria

Table 1 shows glucose levels at the 12 and 20‐week time points in mice with free access to food and water. There was a similar, significant increase in glucose levels in WT and KO Akita mice compared to non‐diabetic WT and KO mice at both time points. In addition, glycated albumin levels were similarly increased in Akita mice compared to non‐diabetic mice at 20 weeks of age (Table 2).

**TABLE 1:** Non‐fasting blood glucose levels (mg/dL).

	Non‐diabetic WT	Non‐diabetic KO	WT Akita	KO Akita
12 Weeks	218 ± 7.2	219 ± 9.9	652 ± 17.5*	666 ± 10.7**
20 Weeks	206 ± 10.5	224 ± 18.6	661 ± 8.9*	638 ± 12.0**

*Note*: *N* = 5 (non‐diabetic WT); *N* = 9 (non‐diabetic KO), *N* = 11 (WT Akita), *N* = 8 (KO Akita). A total of 24 samples were studied.

* *p* < 0.0001 versus non‐diabetic WT.

** *p* < 0.0001 versus non‐diabetic KO.

**TABLE 2:** Glycosylated albumin.

	WT (pmol/mL)	KO (pmol/mL)
Non‐diabetic	14.72 ± 4.57	21.94 ± 1.35
Akia	66.37 ± 12.91*	60.82 ± 9.79**

*Note*: Blood samples were obtained at 20 weeks of age, *N* = 5 (non‐diabetic WT); *N* = 6 (non‐diabetic KO), *N* = 11 (WT Akita), *N* = 9 (KO Akita), A total of 24 samples were studied.

* *p* < 0.025 versus non‐diabetic WT.

** *p* < 0.025 versus Non‐diabetic KO.

**FIGURE 2:**
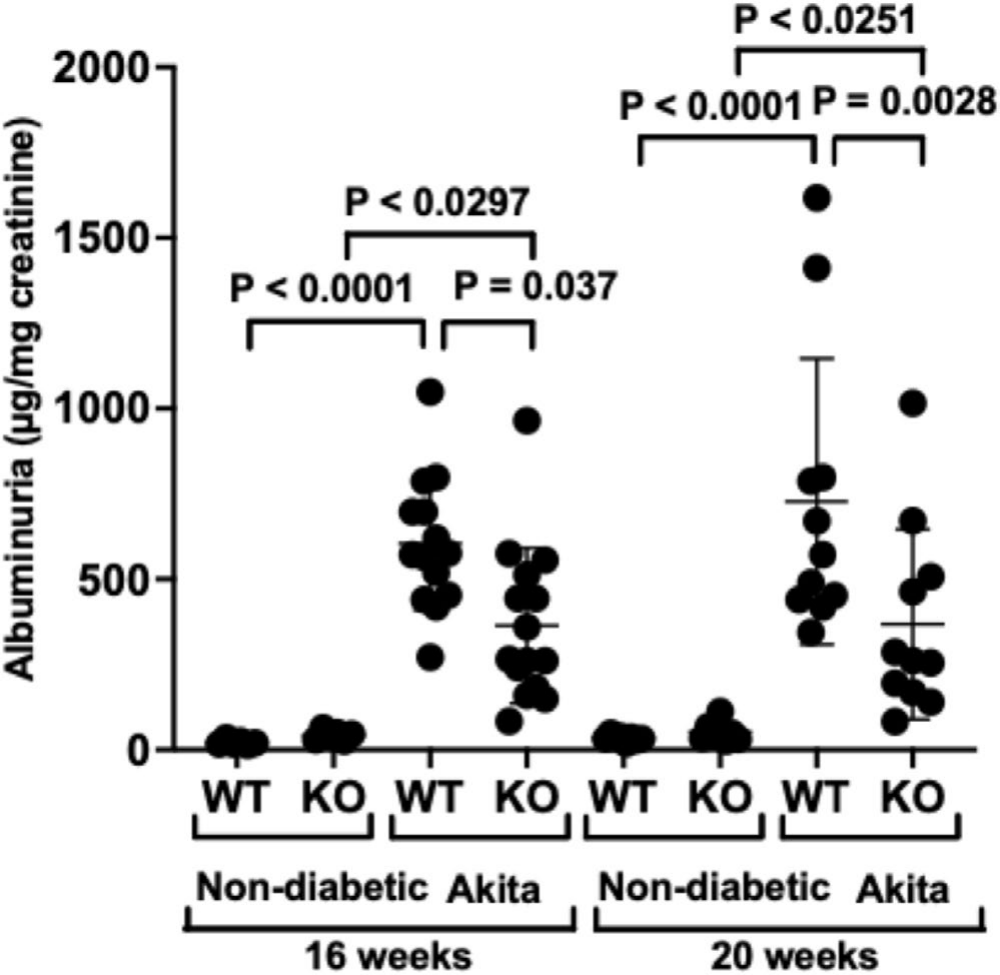
Albuminuria in WT and KO mice. Albuminuria was significantly increased in both WT and KO Akita mice compared to non‐diabetic WT and KO mice. Podocyte specific KO of NPRC significantly reduced albuminuria in KO Akita mice compared to WT Akita mice at both the 16‐ and 20‐week time points. A total of 39 mice were studied at the 16‐week time point, and a total of 36 mice were studied at the 20‐week time point.

Figure 2b shows the effects of NPRC KO on albuminuria in non‐diabetic mice and Akita mice. There was a significant increase in albuminuria in both WT Akita mice and KO Akita mice compared to non‐diabetic WT and KO mice at both the 16‐week and 20‐week time points. In addition, podocyte‐specific KO of NPRC significantly reduced albuminuria in KO Akita mice compared to WT Akita mice at each time point.

### 2.3 Renal histopathology

**FIGURE 3:**
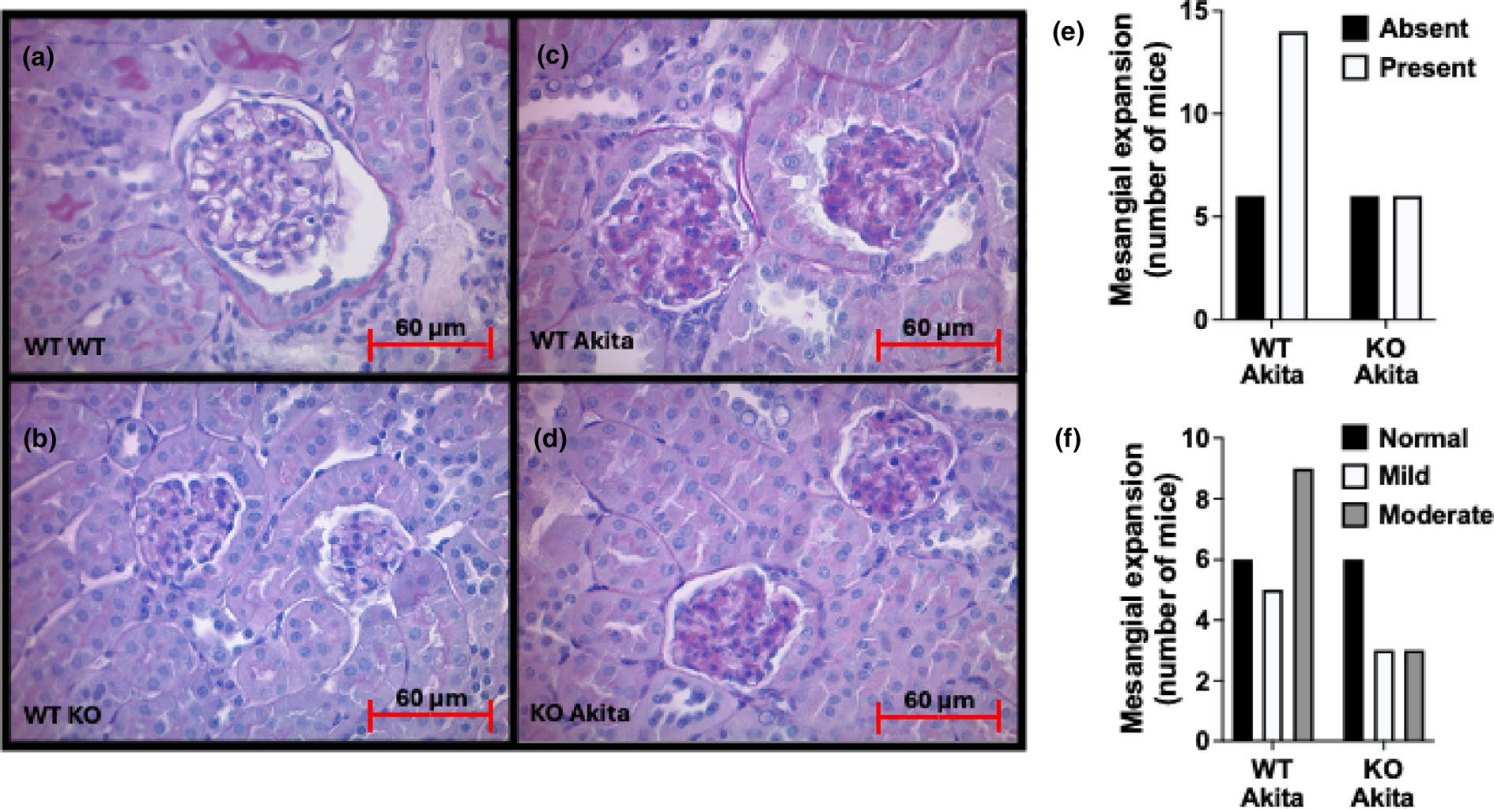
Renal pathology: (a–d). Representative pictures of non‐diabetic and Akita mice. (e) Number of WT‐ and KO‐Akita mice with mesangial expansion. (f) Severity of mesangial expansion in WT‐ and KO‐Akita mice. No mesangial expansion was detected in non‐diabetic mice. These studies were performed using 20‐week‐old mice. A total of 36 kidney specimens were examined for the histologic studies.

Kidney sections were examined by a pathologist without knowledge of the genotype using a previously described qualitative scoring system (Wang et al., 2019) (see Methods). Figure 3a–d show representative pictures of a normal glomerular mesangium in non‐diabetic WT and KO mice (Figure 3a,b), and moderate mesangial expansion in WT and KO Akita mice (Figure 3c,d). Figure 3e,f show the number of Akita mice with increased mesangial matrix and the severity of the mesangial expansion, respectively. Mesangial expansion was detected in 14 of 20 (70%) WT Akita mice and 6 of 12 (50%) KO Akita mice (Figure 3e). In addition, the severity of mesangial expansion tended to be more severe in the WT Akita mice (Figure 3f), but these differences were not statistically significant (*p* = 0.29). There was no mesangial expansion detected in non‐diabetic WT and KO mice. In addition, there was no tubular injury or tubulointerstitial inflammation detected in either Akita mice or non‐diabetic mice. Lastly, body weights were similarly decreased in WT and KO Akita mice compared to non‐diabetic WT and KO mice; and kidney weights were similarly increased in WT and KO Akita mice compared to non‐diabetic WT and KO mice (Figure S1).

### 2.4 Podocyte numbers and density

Table 3 shows the effects of NPRC KO on podocytes per glomerular profile, glomerular volume and podocyte density. For these studies, results for non‐diabetic WT and KO mice were similar and these data were combined for the studies (controls). Podocyte number was lower in both WT Akita mice compared to KO Akita mice, but these differences were not significantly different. In contrast, glomerular volume was significantly increased in WT Akita mice compared to non‐diabetic controls. Moreover, there was a significant decrease in glomerular volume in KO Akita mice compared to WT Akita mice, and glomerular volume in WT Akita mice was not significantly different from that of non‐diabetic control animals (*p* = 0.2614). As a result, podocyte density was significantly decreased in WT Akita mice compared to controls, and KO of NPRC significantly increased glomerular density compared to the WT Akita mice. (NOTE: Figure S3 shows values for non‐diabetic WT and KO mice).

**TABLE 3:** Podocyte number, volume, and density.

	Podocytes per glomerular profile	Glomerular volume (VGlom μm^3^)	Podocyte density NV (P/glom) (10^6^ μm^3^)
Non‐diabetic controls (*N* = 9)	11.5 ± 1.1	16,062 ± 2321	272.6 ± 44.8
WT Akita (*N* = 9)	10.8 ± 0.8	51,566 ± 9855*	81.0 ± 16.6***
KO Akita (*N* = 9)	10.9 ± 1.6	22,460 ± 9433**	171.2 ± 44.1****

*Note*: Results for non‐diabetic WT and KO mice were similar, and these data were combined for the studies (controls).

* *p* < 0.0001 versus non‐diabetic controls.

** *p* < 0.0001 versus WT Akita mice.

*** *p* < 0.0001 versus non‐diabetic controls.

**** *p* < 0.0001 versus WT Akita mice.

### 2.5 Expression of fibrotic markers

**FIGURE 4:**
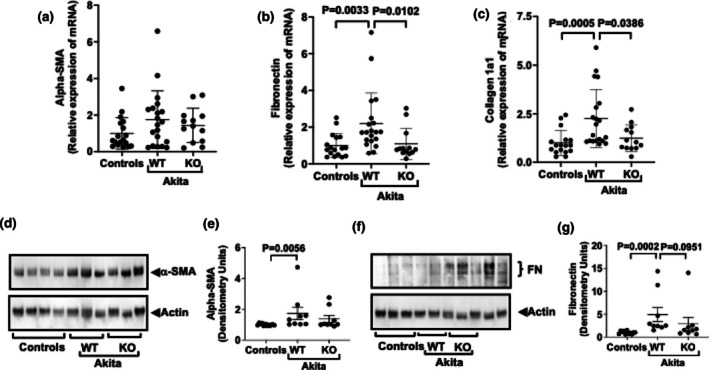
Expression of fibrotic markers: (a–c). There was a significant increase in fibronectin (FN) and collagen 1a1 in WT Akita mice compared to non‐diabetic controls by Q‐RT‐PCR. The increase in fibronectin and collagen 1a1 mRNA was significantly reduced in NPRC‐KO Akita mice compared to WT Akita mice. (d–g). The expression pattern was similar for alpha‐SMA and fibronectin proteins, but the differences were not statistically significant. These studies were performed using 20‐week‐old mice. A total of 50 mRNA samples were used for each Q‐RT‐PCR study. A total of 30 protein samples were used for each immunoblotting study. Results for non‐diabetic WT and KO mice were similar, and these data were combined for the studies (controls).

We next assessed the effect of podocyte specific NPRC KO on expression of the fibrotic markers in glomerular preparations. For the studies, results for non‐diabetic WT and KO mice were similar and these data were combined for the studies (controls). As shown in Figure 4a–c, there was a statistically significant increase in fibronectin and collagen 1 (Cola1) by Q RT‐PCR. In addition, podocyte specific KO of NPRC caused a statistically significant decrease in both collagen 1 and fibronectin. A similar pattern was observed for alpha‐SMA (Figure 4a), but the differences were not statistically different.

Immunoblotting studies detected a statistically significant increase in alpha‐SMA and fibronectin in WT Akita mice, and KO of NPRC tended to decrease expression of both proteins, but these differences were not statistically significant. (NOTE: Figure S4 shows values for non‐diabetic fibrotic markers in WT and KO mice, and Figures S6 and S7 show unedited immunoblots of fibrotic markers.).

### 2.6 Nephrin and podocin expression

**FIGURE 5:**
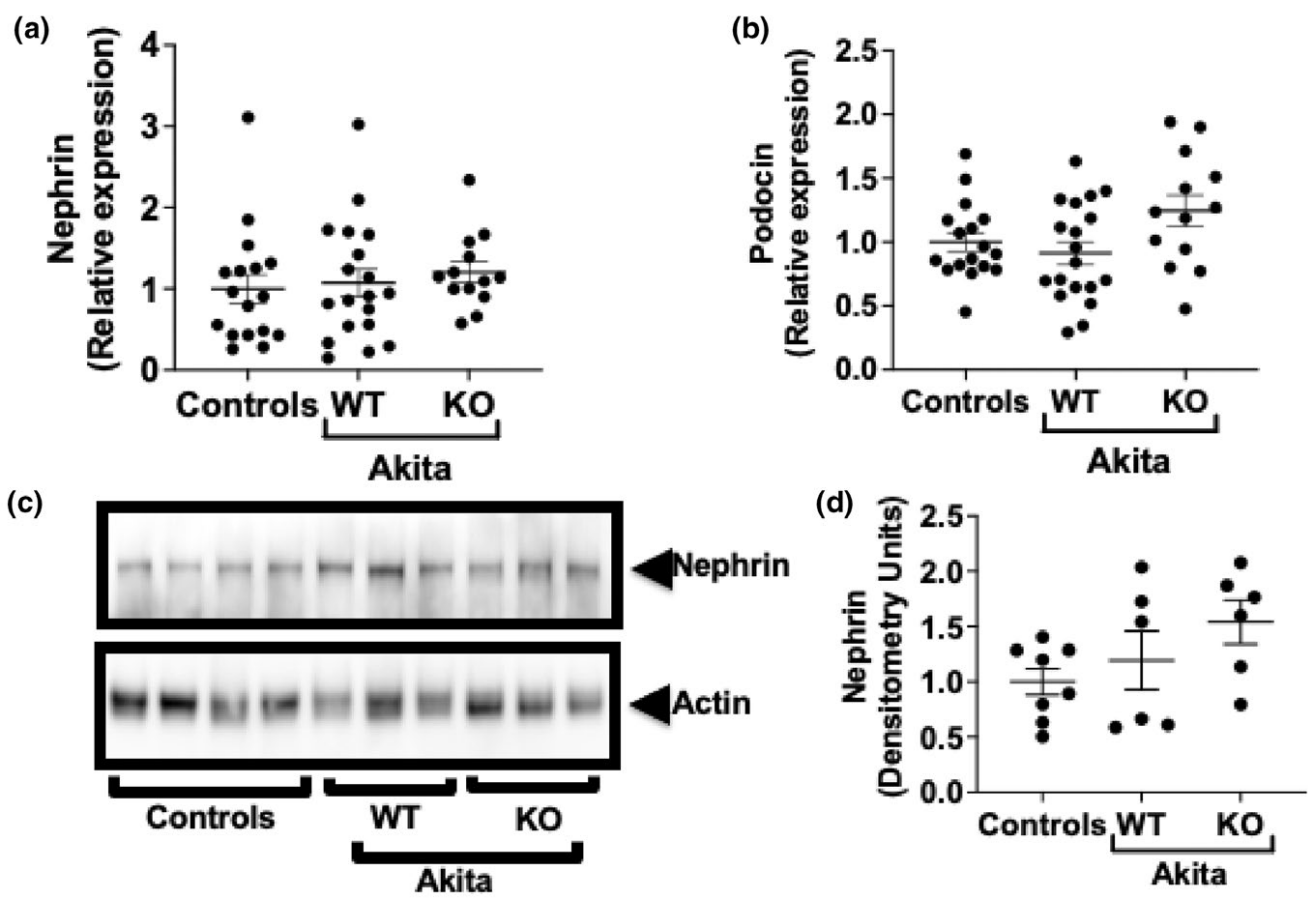
Expression of nephrin and podocin. (a) Expression of nephrin mRNA was similar in WT Akita mice, KO Akita mice, and non‐diabetic controls, and no statistically significant differences were noted between the groups. (b) There were no statistically significant differences in podocin mRNA expression in WT Akita mice, KO Akita mice, or non‐diabetic controls. (c, d) There were no statistically significant differences in expression of nephrin protein between WT Akita mice, KO Akita mice, or non‐diabetic controls. These studies were performed using 20‐week‐old mice. A total of 28 mRNA samples were used for each Q‐RT‐PCR study. A total of 20 protein sampled were used for each immunoblotting study. Results for non‐diabetic WT and KO mice were similar, and these data were combined for the studies (controls).

To determine if podocyte injury was altered in Akita mice, we examined nephrin and podocin levels using both quantitative RT‐PCR and immunoblotting. For these studies, data for non‐diabetic WT and KO mice were similar and these data were combined for the studies (controls). As shown in Figure 5a–d, no statistically significant differences were observed in expression of either nephrin or podocin by either Q‐RT‐PCR or immunoblotting (Figure 5a–d). There were also no significant differences in expression of either synaptopodin or WT1 (Wilms tumor 1) in Akita mice compared to controls by quantitative RT‐PCR (Figure S2). (NOTE: Figure S5 shows values for nephrin and podocin in non‐diabetic WT and KO mice, and Figure S8 shows images of unedited immunoblots of nephrin.).

## 3 DISCUSSION

Recent studies suggest that NPs have protective effects in kidney disease models (Makino et al., 2006; Ogawa et al., 2012; Shen et al., 2016; Staffel et al., 2017; Suganami et al., 2001; Wang et al., 2021; Wang, Tang, et al., 2025). To enhance the beneficial actions of NPs, we deleted NPRC specifically in glomerular podocytes in a mouse model of type 1 diabetes. The goal of this approach was to increase local NP levels and augment the podocyte protective actions of NPs. In support of this approach, we previously found that podocyte specific KO of NPRC successfully attenuated glomerular injury in a mouse model of FSGS (Wang, Tang, et al., 2025). Using diabetic Akita mice, we found that DKD caused a significant increase in albuminuria and increased expression of the extracellular matrix (ECM) proteins collagen‐1 and fibronectin. KO of NPRC significantly reduced albuminuria and inhibited expression of both fibrotic markers (collagen‐1 and fibronectin). These data suggest that KO of NPRC had beneficial effects on both proteinuria and activation of fibrotic pathways in a mouse model of early stage DKD.

To investigate the beneficial effects of NPs in the kidney, we focused treatment on glomerular podocytes because: (1) Podocytes play a key role in the development of DKD (Susztak et al., 2006; Wiggins, 2007; Wolf et al., 2005), and (2) High levels of NPRC are expressed in podocytes compared to other types of kidney cells (Park et al., 2018; Staffel et al., 2017; Wilson et al., 2019). The effects of NPs are, however, regulated by multiple mechanisms including: (1) The endoprotease neprilysin that cleaves NPs (Potter et al., 2006), and (2) Phosphodiesterases (PDEs) that inhibit intracellular signaling of NPs by cleaving cyclic‐GMP (Potter et al., 2006). In addition, both neprilysin and the cGMP‐specific PDEs (PDE5 and PDE9) are expressed in multiple cell types in the kidney including glomerular podocytes (Debiec et al., 2002; Dousa, 1999; Maurice et al., 2014; Park et al., 2018; Wang et al., 2021; Wilson et al., 2019). To determine the most effective approach to stimulate NP‐induced cGMP generation, we previously compared the effects of pharmacologic blockade of NPRC with inhibition of either neprilysin, PDE5, or PDE9 using effective doses of each inhibitor. These studies found that pharmacologic blockade of NPRC was the most effective approach to stimulate NP signaling (Wang et al., 2021). Consistent with our cell culture studies, animal experiments found that NP levels were increased to a greater extent by inhibiting NPRC versus inhibiting neprilysin (Charles et al., 1996; Hashimoto et al., 1994; Okolicany et al., 1992; Potter et al., 2006; Wang et al., 2021). These findings indicate that pharmacologic blockade of NPRC is an effective approach to promote NP signaling.

In addition, NP signaling inhibits multiple signaling pathways that promote kidney injury. For example, NP signaling: (1) Suppresses secretion of endothelins (Glenn et al., 2009; Kohno et al., 1991), (2) Blocks renin release from the juxtaglomerular apparatus (Kurtz et al., 1986) and (3) Reduces calcium signaling by both inhibiting ion channels such as TRPC6 and attenuating calcium release from intracellular stores (Cornwell et al., 1991; Francis et al., 2010; Kinoshita et al., 2010; Nishida et al., 2010; Takahashi et al., 2008). In addition, NPs have important effects on fibrotic signaling. For example, NPs phosphorylate smad3 on an inhibitor site and reduce fibrotic signaling by TGF‐beta (Shen et al., 2016). Similarly, NPs inhibit signaling by RhoA and, in turn, reduce transcription of genes that promote fibrosis including connective tissue growth factor (CTGF), alpha‐SMA and collagen proteins (Shen et al., 2016; Tsou et al., 2014). In support of an important role of NPS in inhibiting fibrosis, a recent study found that enhancing the effects of NPs had beneficial effects on renal fibrosis and improved renal function (Wang, Li, et al., 2025). These data suggest that the beneficial effects of NPRC KO in kidney disease are mediated by multiple mechanisms.

In addition to inhibiting pathways that cause kidney injury, NPRC is also reported to act as a signaling receptor that activates phospholipase C and inhibits both L‐type ion channels and cAMP generation (El Andalousi et al., 2013; Li et al., 2014; Murthy et al., 1998). This signaling pathway has been studied in the heart (Khambata et al., 2011; Li et al., 2014), but little is known about the effects of this signaling cascade in the kidney. Additional studies will be necessary to determine if the effects of NPRC signaling in the kidney contribute to modulating the severity of kidney disease.

An important consideration in evaluating the results of this study is the mild severity of DKD in the C57BL/6 Akita model. Unfortunately, most mouse models of DKD do not have a robust phenotype (Brosius 3rd et al., 2009; Soler et al., 2012) including several Akita models that have been closely examined (Gurley et al., 2009; Chang et al., 2012; Chang & Gurley, 2012; Gurley et al., 2006). In the current study, the difference between non‐diabetic WT mice and WT Akita mice was modest, and there was variability in the phenotypes of both WT Akita mice and KO Akita mice. In addition, decreased expression of the podocyte proteins nephrin and podocin is closely linked to the development of glomerular injury and, in turn, structural damage to the filtration barrier and proteinuria (Aaltonen et al., 2001; Haley et al., 2018; Kondapi et al., 2021; Toyoda et al., 2004). While structural damage to the glomerulus affects size selectivity and promotes albuminuria, electrical charge also plays a significant role in regulating proteinuria (D'Amico & Bazzi, 2003; Lewis & Xu, 2008). Negatively charged molecules (such as albumin) are less readily filtered compared to neutral or positively charged proteins of the same size due to the negatively charged proteins in the glomerular filtration barrier (D'Amico & Bazzi, 2003). Kidney disease promotes loss of negatively charged heparan sulfates in the glomerular filtration barrier and promotes albuminuria (D'Amico & Bazzi, 2003). As a result, a loss of negative charges in the glomerular filtration barrier plays an important role in promoting albuminuria, especially in the early stages of DKD.

An unexpected finding in the current study was the differences in glomerular volume in WT and KO Akita mice. It is well known that diabetes induces hyperfiltration and glomerular hypertrophy (Moriconi et al., 2023; Osterby & Gundersen, 1975), but we were surprised that NPRC KO in Akita mice decreased glomerular volume (reduced hypertrophy). Because podocytes have a limited ability to proliferate (Kriz et al., 1998; Wiggins, 2007), an increase in glomerular volume decreases podocyte density (Haruhara et al., 2024; Minakawa et al., 2019). To compensate for increased glomerular volume, podocytes hypertrophy, but excessive hypertrophy and continued hyperfiltration are postulated to promote podocyte damage leading to proteinuria, podocyte loss, and glomerulosclerosis (Dalla Vestra et al., 2003; Meyer et al., 1999; Minakawa et al., 2019; Teiken et al., 2008; Tsuboi et al., 2010). In this scenario, decreasing glomerular density both increases the risk of podocyte damage and accurately predicts disease progression (Dalla Vestra et al., 2003; Meyer et al., 1999; Minakawa et al., 2019; Teiken et al., 2008; Tsuboi et al., 2010) better than changes in podocyte number (Haruhara et al., 2024). While additional studies are required, we speculate that the differences in podocyte density in WT and KO Akita mice may have contributed to the beneficial outcomes in KO Akita mice.

Lastly, the effects of natriuretic peptides on chronic kidney disease (CKD) have also been investigated in several human trials using a drug (Entresto) composed of the angiotensin receptor blocker valsartan, and the neprilysin inhibitor sacubitril (inhibits cleavage of NPs). This drug combination has been studied in two secondary analyses of patients with impaired cardiac function, which found a reduction in the rate of decline in kidney function (Haynes et al., 2018; Mc Causland et al., 2020; Packer et al., 2020). Interpretation of these studies is, however, limited by the beneficial effects of the drug on heart disease, which may have improved renal hemodynamics. An additional randomized, double‐blind study compared the effects of an angiotensin receptor blocker with sacubitril/valsartan (Haynes et al., 2018). This study found an improvement in hypertension in the sacubitril/valsartan group, but no significant improvement in renal function. This study, however, is difficult to interpret due to the low number of patients with glomerular disease and the short study period. As a result, additional human studies will be necessary to better assess the effects of augmenting the actions of NPs in human trials.

In summary, we knocked down NPRC in podocytes to enhance the podocyte protective actions of NPs. We found that podocyte specific KO of NPRC had beneficial effects on proteinuria and expression of fibrotic markers in Akita mice. These data suggest that inhibiting NP clearance by NPRC might be a useful therapeutic approach to treat DKD and, perhaps, other glomerular diseases.

## 4 METHODS

### 4.1 Materials

Primary antibodies used for the studies included: (1) A mouse monoclonal antibody to alpha‐smooth muscle actin (Durand‐Arczynska et al., 1993) (clone 1A4, catalog number: A5228, Sigma‐Aldrich, St. Louis, MO), (2) A mouse monoclonal antibody to alpha‐actin (Lessard, 1988) (clone C4, catalog number: MA1501, Sigma‐Aldrich, St. Louis, MO), (3) A rabbit polyclonal antibody to fibronectin (catalog number: ab2413, Abcam biotechnology, Cambridge, United Kingdom), and (4) A goat polyclonal antibody to nephrin (Wong et al., 2018) (catalog number: AF3159, R&D Systems, Minneapolis, MN). Secondary antibodies used for the studies included: (1) A mouse HRP‐linked anti‐goat polyclonal antibody (catalog number: sc‐2354, Santa Cruz Biotechnology, Dallas, TX), (2) An anti‐mouse HRP‐linked polyclonal antibody (catalog #: 7076) and (3) An anti‐rabbit HRP‐linked polyclonal antibody (catalog #: 7074) from Cell Signaling Technology, Danvers, MA. Additional materials included: (1) Albuwell (Catalog No. 1011. Ethos Biosciences, Logan Township, NJ), (2) Creatinine Companion kits (catalog No. 1012 Ethos Biosciences), and (3) ELISA kit from AGF Scientific (AFG Bioscience LLC, Northbrook, Illinois).

### 4.2 Animal housing

Mice were housed in groups of 2–4 animals per ventilated cage at 72°F (22.2°C) in a 12‐h light–dark cycle. Food was obtained from Purina Lab Diets, numbers 5001 (regular mouse diets) and 5015 (high fat diet for breeding/weaning). Water was provided in bottles using a triple filtered automatic system.

### 4.3 Creation of NPRC KO mice

The following C57BL/6 mice were obtained for the studies: (1) C57BL/6 mice with 2 “floxed” alleles flanking exon 3 of NPRC (NPRC flox/flox mice) were obtained from Taconic Biosciences (Model No. 13105), (2) C57BL/6 mice that express a single allele of Cre recombinase under the regulation of the podocyte specific podocin promoter (NPHS2‐Cre mice) (Shigehara et al., 2003), from Jackson Labs (Stock No: 008205) and 3. A mouse model of type 1 diabetes (C57BL/6 Akita mice), which was a gift of Dr. Susan Gurley (50, 52). Akita mice have a mutation in the insulin‐2 gene and spontaneously develop type 1 diabetes. These mice (Akita mice) express a single allele of the Akita gene because animals that carry 2 alleles have severe disease and a short life span (Yu et al., 2012).

To generate the mice for the studies, we bred the mouse lines mentioned above by first crossing mice with 2 “floxed” alleles of NPRC with mice expressing Cre recombinase to create NPRC flox/WT mice expressing Cre recombinase. Next, we crossed mice with 2 “floxed” alleles of NPRC with Akita mice to create NPRC flox/WT mice expressing the Akita allele. We then crossed NPRC flox/WT mice expressing Cre recombinase with NPRC flox/WT mice expressing the Akia gene to create the following genotypes:

- *Non‐diabetic WT mice*: (1) NPRC flox/flox mice lacking both the Akita gene and Cre recombinase, (2) NPRC flox/WT mice lacking both the Akita gene and Cre recombinase, or (3) NPRC WT/WT mice expressing Cre recombinase but lacking the Akita gene;
- *Non‐diabetic KO mice*: NPRC flox/flox mice expressing Cre recombinase but lacking the Akita gene;
- *WT Akita mice*: (1) NPRC WT/WT mice expressing both the Akita gene and Cre recombinase, (2) NPRC flox/WT mice expressing the Akita gene but lacking Cre recombinase, or (3) NPRC flox/flox mice expressing the Akita gene but lacking Cre recombinase;
- *KO Akita mice*: NPRC flox/flox mice expressing both the Akita gene and Cre recombinase

### 4.4 Podocyte specific NPRC KO in Tdtomato mice

To assess NPRC KO, we used a reporter mouse available from Jackson Labs (Stock Nos. 007576) (Wang et al., 2017). The reporter construct contains the Tdtomato sequence and stop site flanked by LoxP sites followed by the enhanced green fluorescence protein (EGFP) sequence and a stop site (Muzumdar et al., 2007). Prior to Cre expression, the reporter mice express a cell membrane‐localized red fluorescence protein (Tdtomato) with widespread expression in all tissues and cell types. Expression of Cre recombinase deletes the Tdtomato sequence and stop site and induces expression of EGFP (enhanced green fluorescence protein) only in cells expressing Cre recombinase. For the studies, Tdtomato reporter mice were crossed with mice expressing both a podocyte‐specific Cre recombinase and LoxP sites flanking exon 3 of NPRC to create either: (1) Tdtomato mice expressing a podocyte‐specific Cre recombinase (NPRC+/+ reporter mice) or (2) Tdtomato mice expressing both a podocyte‐specific Cre recombinase and LoxP sites flanking exon 3 of NPRC (NPRC−/− reporter mice). In these mice, both WT and KO reporter mice are labeled with EGFP, but NPRC is deleted only in KO reporter mice.

### 4.5 BP measurements

Systolic BP was measured using a computerized tail‐cuff system (Hatteras Instruments, Cary, NC, USA) in conscious mice as previously described [84]. To reduce variability in the results, mice were acclimated to the experimental conditions for a week prior to the BP measurements. This technique has previously been shown to correlate closely with intra‐arterial measurements (Whitesall et al., 2004).

### 4.6 Glycated albumin

Glycated albumin levels were measured using an ELISA kit (see Materials) and serum from 20‐week‐old mice according to the manufacturer's recommendations.

### 4.7 Histopathology

Light microscopic sections were stained with hematoxylin and eosin (H&E) and periodic acid Schiff (PAS). The slides were then evaluated by a pathologist (A.F.B.) blinded to genotype. Twenty‐five glomeruli were evaluated in each tissue section to assess the average severity of mesangial expansion. Tubules and tubulointerstitial (TI) areas were examined for tubule injury, dilation and casts, and TI inflammation and fibrosis. No significant TI fibrosis was present in diabetic mice; so, this score was based on the severity of inflammation as described below. Abnormalities were graded using a semi‐quantitative scale of 0–3 (0‐normal, 1‐mild, 2‐ moderate, 3‐severe) as previously described (Wang et al., 2019) based on the following criteria:

#### 4.7.1 Mesangial expansion

1. Normal (baseline): Mesangial matrix occupies <10% of glomerular tuft area
2. Mild: 10%–25% of the glomerular tuft area
3. Moderate: >25%–50% of the glomerular tuft area
4. Severe: >50% of the glomerular tuft area

#### 4.7.2 Tubule injury

1. Normal (baseline): None to minimal tubular dilation and casts without tubular degeneration or regeneration
2. Mild: Tubular degeneration and regeneration with/without tubular dilation and casts, involving <10% of cortex
3. Moderate: Tubular degeneration and regeneration with/without tubular dilation and casts, involving 10%–25% of cortex
4. Severe: Tubular degeneration and regeneration with/without tubular dilation and casts, involving >25% of cortex

#### 4.7.3 TI inflammation

1. Normal (baseline): One focus of inflammation (up to 15 mononuclear cells)
2. Mild: Two foci of 15+ mononuclear cells or 1 focus of 30+ mononuclear cells involving up to 5% of area of cortical parenchyma
3. Moderate: Inflammation involving >5%–25% of the cortical parenchyma
4. Severe: Inflammation involving >25% of the cortical parenchyma

### 4.8 Animal experiments

Animal experiments were performed using C57BL/6 mice and the following groups: (1) WT Akita mice, (2) KO Akita mice lacking NPRC specifically in podocytes, (3) Non‐diabetic WT mice, and 4. Non‐diabetic KO mice lacking NPRC specifically in podocytes. Non‐fasting blood glucose levels were measured at 12 and 20 weeks of age using the AlphaTRAK 2 testing system (Abbott Laboratories, Chicago, IL) calibrated for glucose measurements in mice according to directions of the manufacturer. Twenty‐four‐hour urine collections were obtained at 16 and 20 weeks of age using metabolic cages specifically designed for collection of mouse urine (Hatteras Instruments, Cary, NC). After the last urine collection, blood was obtained and then mice were sacrificed and kidneys removed. Kidneys were weighed and kidney tissue was saved in formalin for light microscopic examination.

### 4.9 Quantitation of glomerular volume, podocyte number, and podocyte density

The mean glomerular volume (VGlom) was obtained from the arithmetic mean of the glomerular surface area using the method of Weibel and the equation described by Gundersen and coworkers (Hirose et al., 1982), as previously described (Wang et al., 2015). To quantify the number of podocytes per glomerular profile, frozen kidney cortices were stained with a rabbit polyclonal antibody to WT1 conjugated to Alexa Fluor 488 (Abcam, No. ab89901), and a mouse monoclonal antibody to synaptopodin conjugated to fluorescein (Progen Biotecnik, catalog number 65194). Nuclei were counterstained with DAPI (4′,6‐diamidino‐2‐phenylindole) as previously described (Wang et al., 2015). Podocytes were then quantified by counting cells that both co‐localized with DAPI and were associated with synaptopodin staining. Using this technique, staining was consistently associated with synaptopodin‐stained cells. Podocyte number per glomerulus was quantified using the methodology described by Wiggins and coworkers (Venkatareddy et al., 2014) by converting the number of podocytes per glomerular profile into a podocyte density [Nv(P/Glom)] using an Excel spreadsheet supplied as supplementary data to the manuscript by Wiggins and colleagues (Venkatareddy et al., 2014). For the studies, all available glomeruli were evaluated in each frozen tissue section.

### 4.10 Glomerular isolation

Kidneys were harvested and placed in ice‐cold D‐PBS. The inner medulla was removed, and the cortex was minced and mechanically dissociated by passing through a 180 μm sieve. The resulting cell suspension was collected, further dispersed by passing through a 22‐gauge needle in a 10 cc syringe, and then passed through a 70 μm sieve. The sieve was rinsed thoroughly with cold D‐PBS, and the retained glomeruli were collected and frozen at −80°C. Frozen samples were later used to either: (1) Solubilize proteins for immunoblotting, or (2) Prepare mRNA for quantitative RT‐PCR or immunoblotting.

### 4.11 Flow cytometry

Freshly isolated mouse glomeruli (see Glomerular Isolation) were subjected to enzymatic digestion by suspending glomeruli in 1 mL of digestion buffer (Liberase TM, DNase I, and Hyaluronidase in HBSS) and digested for 30 min at 37°C in a rotating shaker. After centrifugation and red blood cell lysis, the pellet was treated with 0.25% Trypsin supplemented with DNase I for 10 min at 37°C. The reaction was quenched with PBS containing 10% FBS, and the suspension was pipetted vigorously to prevent clumping. Cells were then washed sequentially with PBS containing 10% FBS and PBS containing 0.01% BSA before a final filtration through a 40 μm strainer to generate a single‐cell suspension for staining and flow cytometry analysis. For the flow cytometry studies, kidney cortices from 4 mice in each group were combined in order to collect a sufficient number of podocytes for the studies.

### 4.12 Immunoblotting of enriched glomerular preparations

Immunoblotting was performed using the antibodies listed in Materials. Briefly, enriched glomerular pellets were solubilized by sonication in NP‐40 lysis buffer (50 mM Tris–HCl, 150 mM sodium chloride, 2 mM ethylenediaminetetraacetic acid, 1% NP‐40) and protease inhibitors (Sigma‐Aldrich, St. Louis, MO, catalog number P8340), and frozen at −80°C until the time of study. Proteins were separated using the XCell SureLock Bis‐Tris Mini‐Cell Electrophoresis System (Thermo Fisher Scientific, Waltham, MA) and transferred to PVDF (polyvinylidene difluoride) membranes (Thermo Fisher, Catalog No. LC2002) according to the directions of the manufacturer. Immunoblots were then blocked in 20 mM Tris–HCl, 137 mM NaCl, pH 7.6 (TBS) with 0.2% Tween 20 (T‐TBS) and 2% bovine serum albumin(BSA, (Sigma‐Aldrich, St. Louis, MO, Catalog No. 05470‐5G)). The primary antibody was added at a 1:1000 dilution in blocking buffer and incubated overnight. After washing, the HRP (horseradish peroxidase) linked secondary antibodies were added at a 1:2000 concentration in blocking solution and incubated for 1 h prior to washing. Protein detection was performed using enhanced chemiluminescence (ECL) (Thermo Scientific, Waltham, MA, Catalog number. WP20005) according to the directions of the manufacturer. Imaging of Western blots was performed using a Bio‐Rad ChemiDoc‐MP imaging system. To assess protein‐loading immunoblots were stripped using Restore Western Blot Stripping Buffer (Thermo Scientific, Waltham, MA, catalog number 46430) according to the directions of the manufacturer and immunoblotting was performed using mouse monoclonal antibodies to beta‐actin (0.5 μg/mL) in blocking solution and an HRP‐linked anti‐mouse secondary antibody (1:2000). Densitometry was performed using ScanAnalysis 2.5 software (Biosoft). For the densitometric analyses, the protein signals were divided by the matched signals for beta‐actin. In addition, to compare separate immunoblots, densitometric data were normalized to WT controls. For the immunoblotting studies, results for non‐diabetic WT and KO were similar and these data were combined for the studies (controls).

### 4.13 Expression of glomerular mRNAs

Reverse transcription (RT) followed by a quantitative polymerase chain reaction (Q‐RT‐PCR) was performed using an iCycler TM (Bio‐ Rad Laboratories, Inc., Hercules, CA). For the studies, total cellular RNA was prepared using enriched glomerular preparations and the Trizol reagent according to the manufacturer's directions (Life Technologies Inc., Carlsbad, CA). The RNA was treated with RNase‐free DNase (Sigma‐Aldrich, St. Louis, MO, number 11284932001) and then reverse‐transcribed with iScript Reverse Transcription Supermix (Bio‐Rad, number 1708841) and oligo primers. Real‐time quantitative PCR was performed using an iCycler Q‐PCR machine and the universal SYBR Green PCR Master Mix Kit (Bio Rad, Berkeley, California, number 1708882). The amplification signals were normalized to the endogenous GAPDH mRNA level. The primer sequences used for Q‐RT‐PCR were as follows: NPRC forward, 5′‐AGC TGG CTA CAG CAA GAA GG‐3′ & reverse, 5′‐CGG CGA TAC CTT CAA ATG TC‐3′; GAPDH, 5′_‐GTGAAGGTCGGTGTG AACGGATTTG‐3′ & reverse 5′ ‐ACATTGGGGGTAGGAACACGGAAGG‐3′; Fibronectin forward 5′‐CGA GGT GAC AGA GAC CAC AA‐3′ & reverse 5′‐CTG GAG TCA AGC CAG ACA CA‐3′; collagen type 1, alpha‐1 (COL1A1) forward 5′‐ATC TCC TGG TGC TGA TGG AC‐3′ & reverse 5′‐ACC TTG TTT GCC AGG TTC AC‐3′; alpha‐smooth muscle actin (SMA) forward 5′‐GAG GCA CCA CTG ACC CCT AA‐3′ & reverse 5′‐CAT CTC CAG AGT CCA GCA CA‐3′, Nephrin forward 5′‐AGCTACCCTGCATAGCCAGA‐3′ & reverse 5′‐CCCAAGCTATGGACACTGGT‐3′, Podocin forward 5′‐GTGTCCAAAGCCATCCAGTT‐3′ & reverse 5′‐GCAATGCTCTTCCTTTCCAG‐3′.

### 4.14 Statistical analysis

Data are presented as the mean ± standard deviation and statistical analysis was performed using the Prism 10 computer program (GraphPad Software, Inc.). We first performed a normality test using the Kolmogorov–Smirnov test. If the data failed to pass the normality test, statistical significance was assessed using a Mann–Whitney test for two groups of continuous variables or a Kruskal‐Wallis test for more than 2 groups of continuous variables followed by the Dunn's multiple comparison test. For normally distributed data, we analyzed two groups of continuous variables with a *t*‐test, and for more than 2 groups of continuous variables we analyzed data by an analysis of variance (ANOVA), followed by Sidak's multiple comparisons post‐test. For non‐continuous variables (histopathology), data were analyzed using a Fisher's Exact test using the number of mice with the specified histologic abnormality. All statistics were performed using two‐sided tests.

## AUTHOR CONTRIBUTIONS

**Robert F. Spurney:** Conceptualization; data curation; formal analysis; funding acquisition; investigation; methodology; project administration; resources; supervision; validation. **Liming Wang:** Data curation; formal analysis; investigation; methodology; project administration; supervision. **Yupinp Tang:** Investigation. **Qunsheng Dai:** Formal analysis. **Anne F. Buckley:** Formal analysis; methodology.

## FUNDING INFORMATION

These studies were supported by the following grants: (1) A Merit Review Grant (BX005703) from the Veterans Administration. (2) An R21 grant (TR004257) from the National Institutes of Health.

## CONFLICTS OF INTEREST STATEMENT

The authors declare no conflicts of interest.

## ETHICS STATEMENT

The experiments conformed to the Guide for the Care and Use of Laboratory Animals (S4) and were approved by both the Duke and Veterans Administration Institutional Animal Care and Use Committees.

## Supporting information


Figure S1.

## Data Availability

The data that support the findings of this study are available on request from the corresponding author.
