# Supplementary material for: Podocyte specific knockout (KO) of the natriuretic peptide clearance receptor (NPRC) attenuates diabetic kidney disease (DKD)
Source: Physiol Rep. 2026 May 13;14(9):e70899. doi: 10.14814/phy2.70899 (PMC13172613; doi:10.14814/phy2.70899)

**Supplementary Figure S1**


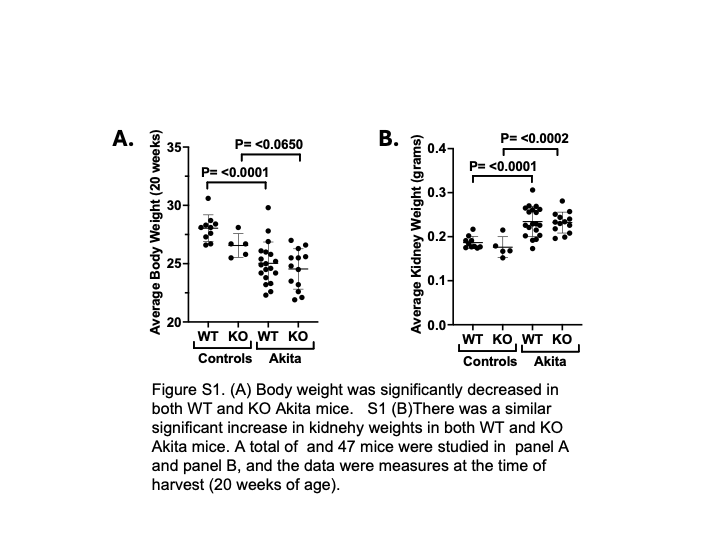


**Supplementary Figure S2**


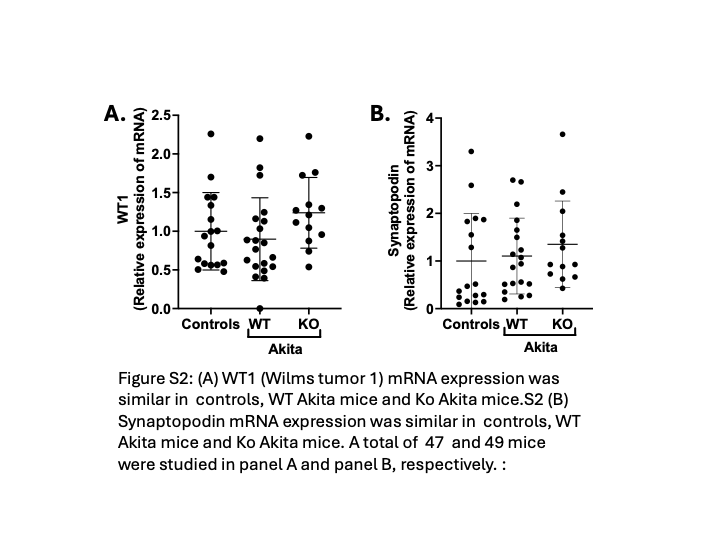


**Supplementary Figure S3.** Podocyte Number , Volume and Density

**Supplementary Figure S4:** Expression of fibrotic markers

**QUANTITATIVE RT PCR**

**IMMUNOBLOTS**

**Supplementary Figure S5:** Expression of nephrin and podocin.

**QUANTITATIVE RT PCR**

**IMMUNOBLOTS**

**Supplementary Figure S6**


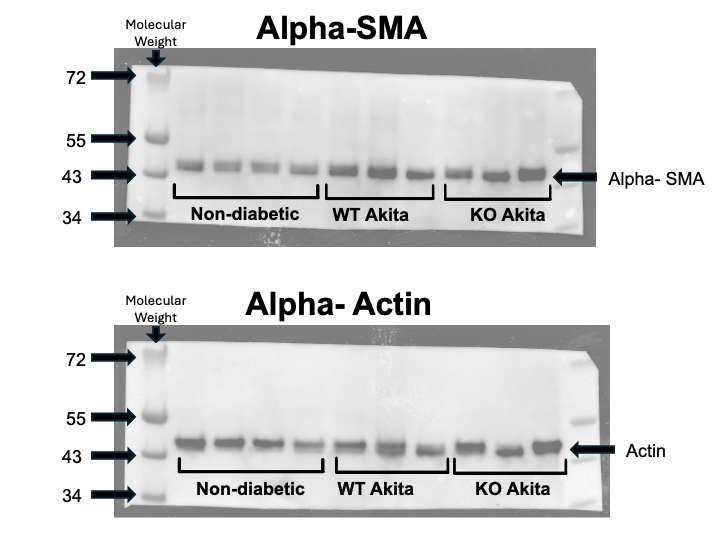


NOTE: The Alpha-SMA blot was stripped and re-probed for alpha-actin.

**Supplementary Figure S7**


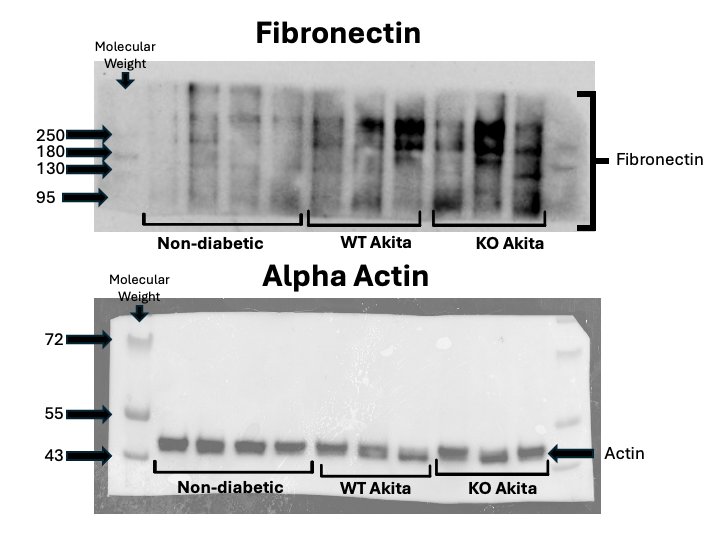


**Supplementary Figure S8**


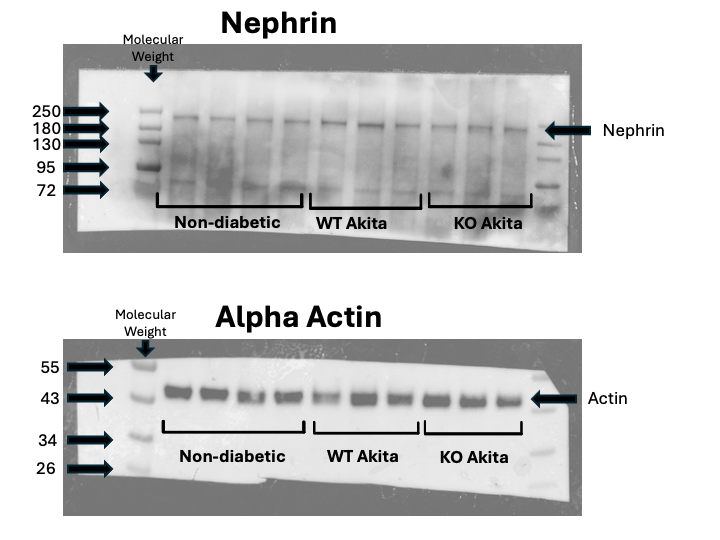

Supplement: Supplementary file 1 — Figure S1. [file PHY2-14-e70899-s001.docx]
